# Biophysical studies and NMR structure of YAP2 WW domain - LATS1 PPxY motif complexes reveal the basis of their interaction

**DOI:** 10.18632/oncotarget.23909

**Published:** 2018-01-03

**Authors:** Apoorva Verma, Fan Jing-Song, Megan L. Finch-Edmondson, Adrian Velazquez-Campoy, Shanker Balasegaran, Marius Sudol, Jayaraman Sivaraman

**Affiliations:** ^1^ Department of Biological Sciences, National University of Singapore, Singapore; ^2^ Mechanobiology Institute, National University of Singapore, Singapore; ^3^ Institute of Biocomputation and Physics of Complex Systems (BIFI)–Joint Units: BIFI–IQFR (CSIC) and GBsC–CSIC, Universidad de Zaragoza, Spain, Department of Biochemistry and Molecular and Cellular Biology, Universidad de Zaragoza, Fundacion ARAID, Gobierno de Aragon, Spain, Aragon Health Research Institute (IIS Aragon), Universidad de Zaragoza, Zaragoza, Spain; ^4^ Department of Physiology, Yong Loo Lin School of Medicine, National University of Singapore, MD9, Singapore; ^5^ Institute of Molecular and Cell Biology (IMCB), Agency for Science, Technology and Research, Singapore

**Keywords:** hippo pathway, YAP, LATS, WW domain, PPxY motif

## Abstract

YES-associated protein (YAP) is a major effector protein of the Hippo tumor suppressor pathway, and is phosphorylated by the serine/threonine kinase LATS. Their binding is mediated by the interaction between WW domains of YAP and PPxY motifs of LATS. Their isoforms, YAP2 and LATS1 contain two WW domains and two PPxY motifs respectively. Here, we report the study of the interaction of these domains both *in vitro* and in human cell lines, to better understand the mechanism of their binding. We show that there is a reciprocal binding preference of YAP2-WW1 with LATS1-PPxY2, and YAP2-WW2 with LATS1-PPxY1. We solved the NMR structures of these complexes and identified several conserved residues that play a critical role in binding. We further created a YAP2 mutant by swapping the WW domains, and found that YAP2 phosphorylation at S127 by LATS1 is not affected by the spatial configuration of its WW domains. This is likely because the region between the PPxY motifs of LATS1 is unstructured, even upon binding with its partner. Based on our observations, we propose possible models for the interaction between YAP2 and LATS1.

## INTRODUCTION

The Hippo tumor suppressor pathway plays a critical role in regulating cell proliferation and apoptosis [1, 2]. First discovered in *Drosophila* [3, 4], the core components of the pathway are highly conserved throughout evolution, with loss-of-function mutants of core pathway components resulting in tissue overgrowth and a diminished cell death phenotype [5]. The pathway has therefore been associated with a wide range of physiological and pathological conditions, including cancer [6], tissue regeneration and wound healing [2]. The Hippo pathway has been extensively reviewed [5, 7–9]. In short, it involves a cascade of phosphorylation events that culminate in the phosphorylation and deactivation of the transcriptional co-activators, YAP and TAZ, by the LATS serine/threonine kinases. Phosphorylated YAP and TAZ are sequestered into the cytoplasm via several known mechanisms [6, 8] to inhibit the transcription of their target genes. Both YAP and TAZ promote cell growth and inhibit cell death, and are thus regarded as potent oncoproteins.

Numerous complexes in the Hippo pathway are mediated by WW domains and their recognition motifs [10]. WW domains are typically 35 to 40 amino acids in length, and are characterized by two highly conserved tryptophan (W) residues separated by 20 to 23 amino acids [11]. They fold into a typical triple-stranded anti-parallel β-sheet and interact with proline-rich motifs, such as PPxY (where P is proline, Y is tyrosine and x is any amino acid) [11]. Despite the conservation in their structure, WW domains within different proteins show ligand-specific interactions [12]. These interactions are further influenced by combinations of tandem WW domains and PPxY motifs in some proteins, which lead to selectivity between partners during signal transduction [13].

The Hippo pathway effector, YAP has two major isoforms, YAP1-1 (abbreviated as YAP1), which has a single WW domain, and YAP1-2 (abbreviated as YAP2), which contains two WW domains separated by a linker of 20 to 25 amino acids [14]. The LATS kinase is also reported to have two isoforms: LATS1, with two PPxY motifs separated by 180 amino acids, and LATS2, with only one PPxY motif [15]. YAP and LATS interact through their WW domains and PPxY motifs, respectively, and mutations in these regions abrogate their binding [16, 17]. However, the importance of these tandem domains and motifs in the specific YAP2 and LATS1 isoforms and the bearing this has on their interaction is not fully understood.

Here, we report the structural, biophysical and cell-based studies of YAP2 WW domains and LATS1 PPxY motifs to understand their interaction. We find that the first WW domain of YAP2 exhibits a preference for the second PPxY motif of LATS1, whereas the second WW domain of YAP2 prefers the first motif of LATS1. We solved the NMR structures of the complexes between WW domains and PPxY-containing peptides, and identified key residues that are important for the interactions. We show that swapping the WW domains of YAP2 has no effect on the phosphorylation status of its highly conserved phosphorylation site, S127, by LATS1. Furthermore, we show that the region between the two PPxY motifs of LATS1 is unstructured, which may regulate its protein-interaction profile. Collectively, these studies widen our understanding of the molecular basis for the interaction between LATS1 and YAP2.

## RESULTS

### YAP2 WW domains exhibit binding preference for LATS1 PPxY motifs

**Figure 1:**
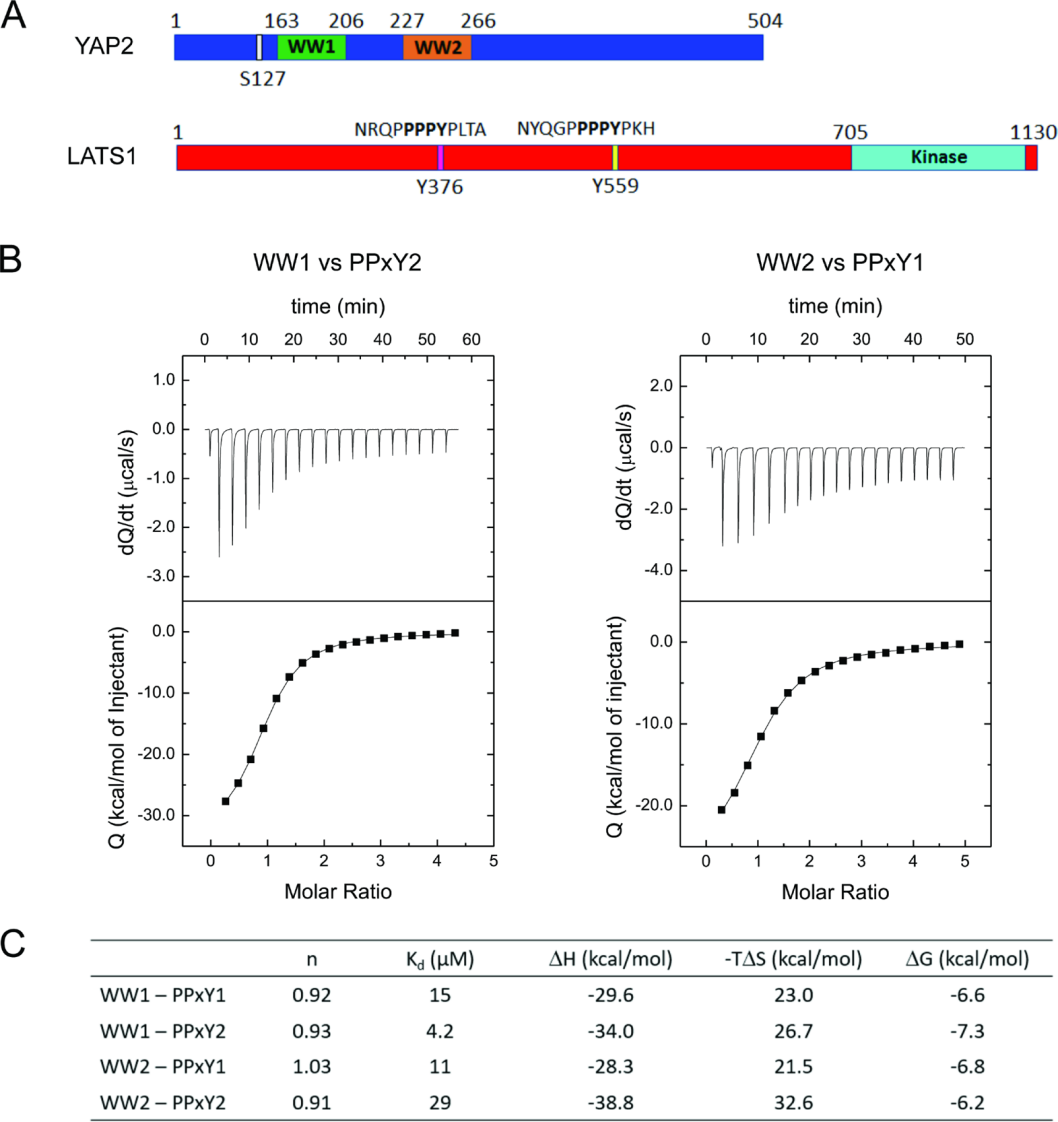
Preference between YAP2 WW domains and LATS1 PPxY peptides (**A**) Schematic representations showing the WW domains from human YAP2 and PPxY peptides from LATS1. (**B**) ITC isotherms for WW1-PPxY2 (left) and WW2-PPxY1 (right) exhibiting the highest binding affinities. (**C**) Summary of thermodynamic parameters for binding among all combinations of YAP2 WW domains and LATS1 PPxY peptides.

We first performed isothermal titration calorimetry (ITC) to test for binding preferences between the two WW domains of YAP2 and the two PPxY motifs of LATS1 (Figure 1A). The individual WW domains (WW1 and WW2) were titrated against peptides containing the PPxY motifs from LATS1, NRQP**PPPY**PLTA (PPxY1) and NYQGP**PPPY**PKH (PPxY2) (Figure 1A).

Among the combinations, the binding affinity was highest between WW1 and PPxY2, with a K_d_ of 4.2 μM (Figure 1B left and 1C). This suggests that WW1 has a binding preference for the second PPxY motif from LATS1. Further, we found that WW2 showed a higher affinity for the first PPxY motif, with a K_d_ of 11 μM (Figure 1B right and 1C) as compared with 29 μM for WW2-PPxY2 or 15 μM for WW1-PPxY1 (Figure 1C, Supplementary Figure 1). A similar observation of preferential binding for YAP2 WW domains with PPxY peptides of other proteins has been reported elsewhere [18]. Previous work has also shown that mutation of either of the domains or motifs weakens the interaction between YAP2 and LATS1, and that mutations of both WW domains [16] or both PPxY motifs [17] completely abolishes the interaction. This suggests that both domains and motifs are involved in mediating the interaction. Based on our ITC results, we suggest that YAP2-WW1 and LATS1-PPxY2 form the basis of the interaction while WW2 and PPxY1 act as additional regulators for their binding.

### NMR structures of WW1-PPxY2 and WW2-PPxY1

Next, to understand the basis of these preferential interactions, we solved the complex structures using NMR. The binding affinity for the complexes was in the micro-molar range and the complex partially dissociated upon injection into gel filtration chromatography (Supplementary Figure 2). Thus, to prevent complex dissociation, we linked the peptides to their respective domains (WW1-PPxY2 and WW2-PPxY1), thereby maintaining homogeneity of the sample. Further, because the complex would be expressed as a single protein, this aided in labelling the peptides with ^15^N and ^13^C.

Poly-glycine linkers can be used to trap weak and transient protein interactions for structural studies [19, 20]. We therefore attempted a range of poly-glycine linkers (of 4 to 8 glycine residues) and compared their gel filtration elution profiles. We anticipated that the complex with an optimum linker length would elute similarly to the unlinked complex. The complex with a 4-glycine linker eluted at the same volume as the unlinked complex, whereas the longer linked constructs eluted at slightly larger volumes (Supplementary Figure 2). The 4-glycine linker was therefore selected for structure determination.

**Figure 2:**
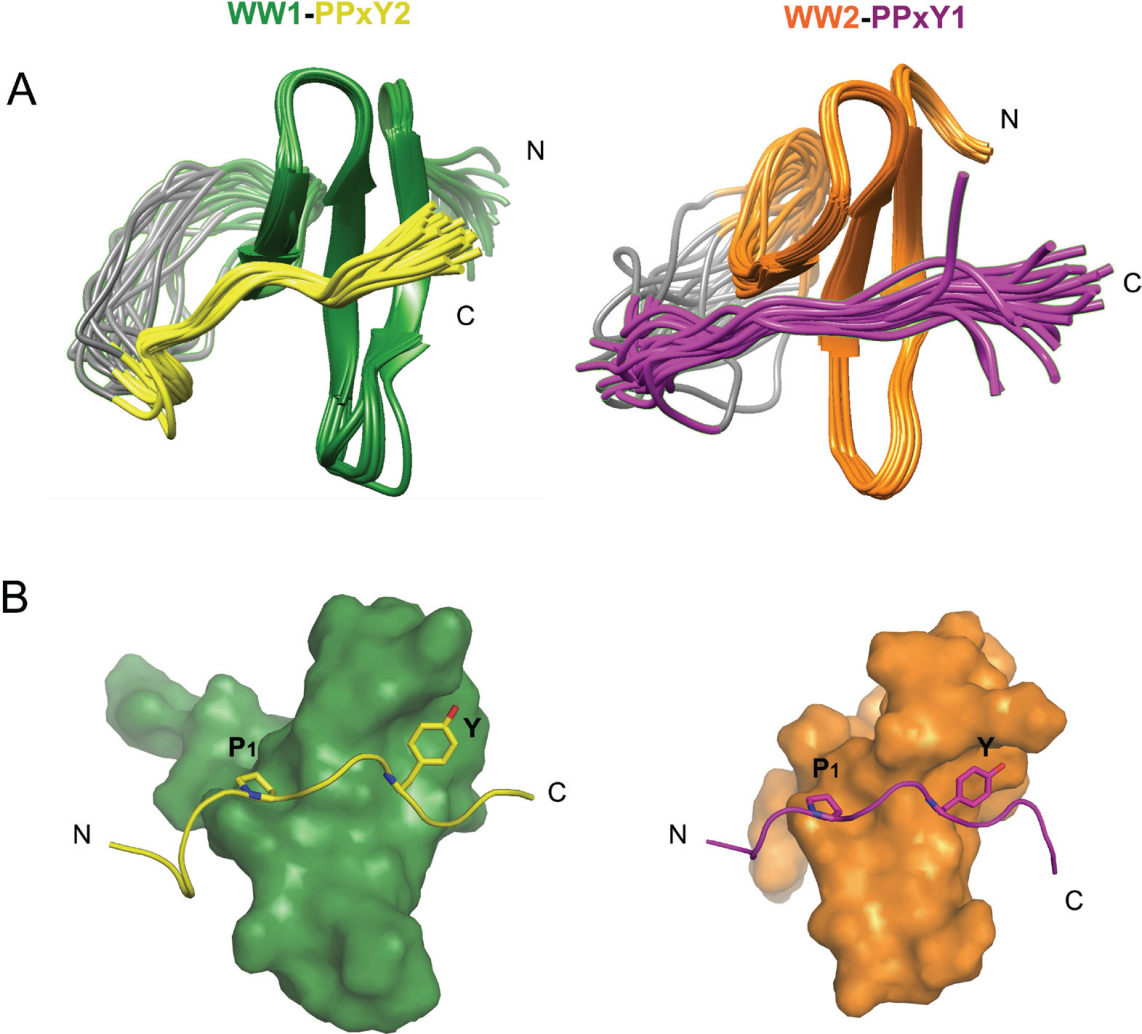
NMR structures of WW1-PPxY2 and WW2-PPxY1 complex (**A**) Structure alignment of the 20 lowest energy structures showing the folded WW domains and unstructured glycine linker holding PPxY peptides interacting with the WW domains (Left: WW1-PPxY2; Right: WW2-PPxY1). (**B**) Molecular surface representation of WW domains bound to the peptides shown in ribbon form. Key interactions of proline (P_1_) and tyrosine (Y) from PPxY motif are shown fitting into their respective pockets in the WW domains (Left: WW1-PPxY2; Right: WW2-PPxY1).

**Figure 3:**
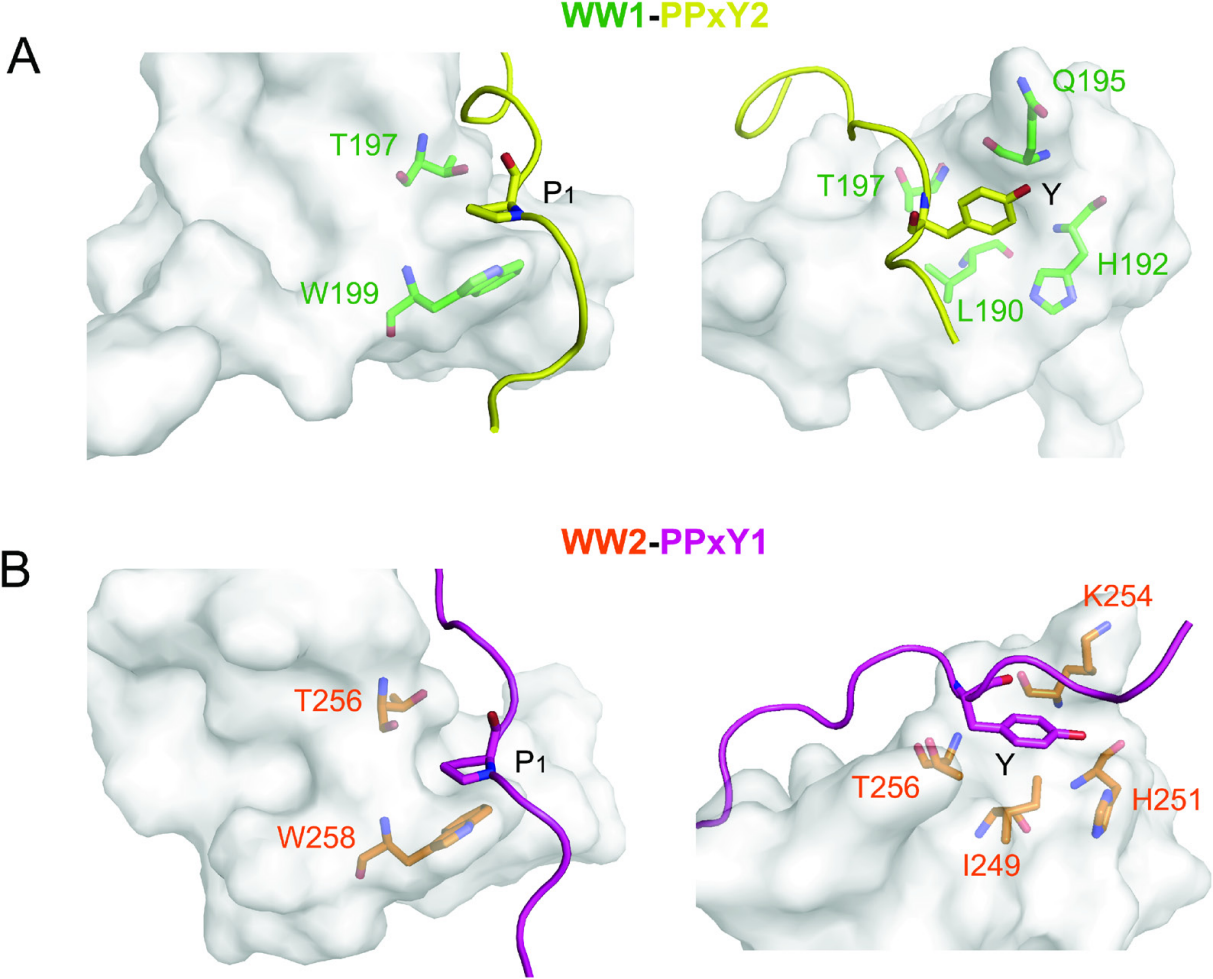
Interactions between WW domains and PPxY peptides (**A**) Interactions of PPxY2 (yellow) motif with WW1 (green) domain showing the P_1_ and Y pockets. P_1_ side chain is sandwiched between W199 and T197, whereas the Y side chain is accommodated in the hydrophobic groove formed by L190, H192, Q195, and T197 from the domain. (**B**) PPxY1 (magenta) motif showing similar interactions with WW2 (orange) domain.

The structures of both linked complexes, WW1-PPxY2 and WW2-PPxY1, were solved using NMR and refined to final backbone RMSD of 0.20Å in the secondary structure region for the 20 lowest energy structures (Table 1). The WW domains exhibit the typical structure comprising of a triple-stranded anti-parallel β-sheet. The glycine linker forms an unstructured loop that holds the peptide in close vicinity to the domain (Figure 2A). The PPxY peptides are present in polyproline type II (PPII) helical conformation. There are two pockets that recognize the PPxY peptide, one for P_1_ and the other for Y (Figure 2B). In the WW1-PPxY2 complex, a total of 41 NOEs were observed between the domain and peptide, among residues corresponding to the two binding pockets. The P_1_ pocket is formed by the side chains of W199 and T197 (Figure 3A left), with a hydrogen bond contact (3Å) between the indole nitrogen of W199 as donor and the backbone nitrogen of P_1_ as acceptor. The Y pocket is a hydrophobic groove composed of side chains from L190, H192, Q195, and T197 that fits the Y side chain from the PPxY peptide (Figure 3A right). The total buried surface area between domain and peptide is 796Å^2^. In the WW2-PPxY1 complex, 25 NOEs were observed between the domain and peptide. The P_1_ pocket is composed of W258 and T256 (Figure 3B left), whereas the Y pocket is formed by I249, H251, K254, and T256 (Figure 3B right), with a total buried surface area of 630Å^2^. It should be noted that the P_1_ and Y pockets in both domains are formed by corresponding residues from each domain, and the difference in buried surface area is consistent with their binding affinities. The two WW domains are 53% identical and 71% similar in sequence. Therefore, both complexes are similar and superimpose with an RMSD of 0.92Å for all Cα atoms in the secondary structure region (Supplementary Figure 3), and are consistent with other known WW structures [21–25].

**Table 1:** Summary of NMR data and structure statistics

	WW1-PPxY2	WW2-PPxY1
**NOE distance restraints *^a^***	998	434
Intra-residue	449	118
Sequential (|*i–j*| = 1)	216	137
Medium range (1 <|*i–j*|<5)	85	44
Long range (|*i–j*|≥5)	248	135
**Hydrogen bond restraints**	16	12
**Dihedral angle restraints (ϕ, ψ) *^b^***	78	38
**Energy statistics (kcal mol^−1^)**		
*E*_noe_	40.475 ± 0.834	14.429 ± 0.561
*E*_cdih_	1.12 ± 0.25	0.122 ± 0.041
**Deviations from idealized covalent geometry**		
R.m.s. deviations of bond lengths (Å)	0.0029 ± 0.00005	0.0022 ± 0.00007
R.m.s. deviations of bond angles (°)	0.445 ± 0.009	0.419 ± 0.009
R.m.s. deviations of improper angles (°)	0.345 ± 0.006	0.313 ± 0.007
**Deviations from experimental restraints**		
R.m.s. deviations of distance restraints (Å)	0.0335 ± 0.0003	0.313 ± 0.007
R.m.s. deviations of dihedral angle restraints (°)	0.483 ± 0.056	0.229 ± 0.04
**Ramachandran plot analysis (%) *^c^***		
Residues in allowed regions	96.8	90.4
Residues in generously allowed regions	3	6.1
Residues in disallowed regions	0.2	3.5
**Average R.m.s. deviations from mean structure (Å) *^d^***		
Heavy atoms	1.12 ± 0.27	0.79 ± 0.15
Backbone atoms	0.20 ± 0.09	0.20 ± 0.06

*^a^* Distance restraints were obtained by classifying the NOE cross peaks into three categories: strong (1.8–2.9 Å), medium (1.8–3.5 Å), and weak (1.8–5.0 Å).

*^b^* Dihedral angles of backbone ϕ and ψ were predicted by TALOS [40] using the chemical shifts of Cα, Cβ, Hα, N, and HN.

*^c^* Calculated with PROCHECK-NMR [42].

*^d^* Calculated with MOLMOL [43] over secondary structure region β1 (177-181), β2 (187-191) and β3 (196-198) for WW1-PPxY2 and β1 (236-240), β2 (246-249) and β3 (255-257) for WW2-PPxY1.

### Additional key residues from WW domains are important for interaction

**Figure 4:**
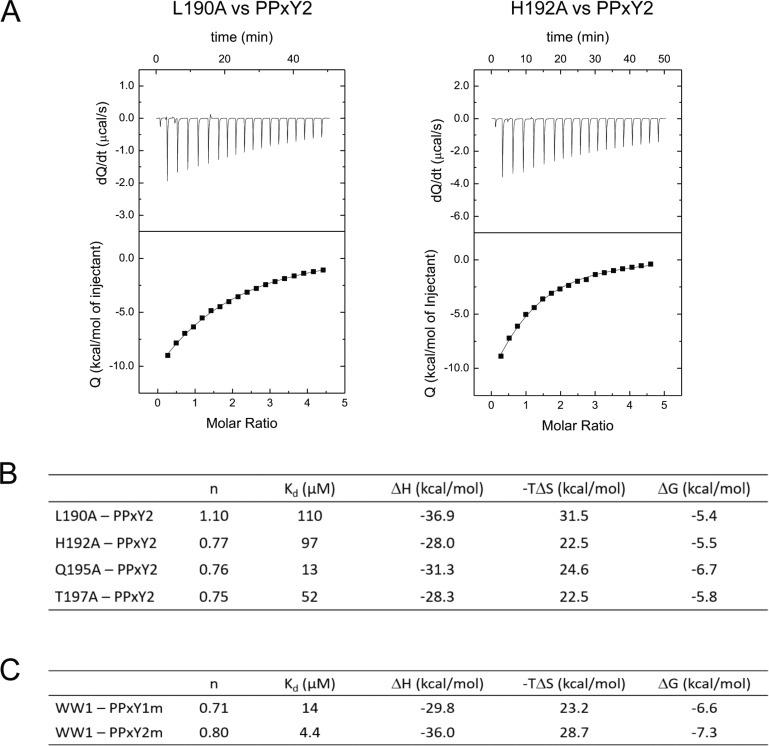
Identification of additional residues from WW domains and PPxY peptides (**A**) ITC isotherms for WW1 mutants, L190A (left) and H192A (right), which displayed maximum decrease in the binding affinity with PPxY2. (**B**) Summary of thermodynamic parameters for binding between all WW1 mutants and PPxY2 peptide. (**C**) Thermodynamic parameters for binding between PPxY2 peptide mutants and WW1 domain.

Since WW1 and PPxY2 exhibited the highest binding affinity, we investigated this complex further. The NMR structure revealed that L190, H192, Q195, T197, and W199 of WW1 interact with the PPxY peptides. Previous studies have shown that W199 is a critical residue in the WW domain, as its mutation abrogates WW-PPxY binding [26]. We thus sought to characterise the role of the additional residues that are involved in mediating the interaction. We mutated each of the aforementioned residues to alanine and performed ITC (Figure 4A and 4B, Supplementary Figure 4). We found that L190A and H192A produced the maximal effect on binding, causing 26- and 23-fold reductions in affinity, respectively, whereas T197A reduced the affinity by 10-fold and Q195A reduced the affinity by 3-fold. Therefore, all these residues are important for the interaction, with L190 and H192 being the most important among them. Similarly, the WW2-PPxY1 structure revealed that these residues are conserved, and mediate similar interactions with PPxY1.

### Role of flanking residues of the PPxY motif in recognition

In both cases, the LATS1 PPxY peptides contain the same ‘PPPPYP’ sequence, differing only by the flanking residues. Yet, these two peptides exhibit different binding affinities to the two WW domains. This suggests that flanking residues might play an important role in mediating peptide recognition. Based on our structures, we observed that R from PPxY1 (P_1_-3) and Q from PPxY2 (P_1_-3) make contact (3 to 3.5 Å) with the WW domains. We subsequently mutated each of these residues to alanine to examine their roles in binding. ITC experiments were performed with WW1, as it showed the highest affinity in previous experiments. In the case of PPxY1_R→A_, the K_d_ changed from 15 μM to 14 μM, whereas the K_d_ of PPxY2 _Q→A_ changed from 4.2 to 4.4 μM (Figure 4C, Supplementary Figure 5). As the observed change is negligible, it is likely that other residues might be involved in the recognition.

### Swapping the YAP2 WW domains does not affect S127 phosphorylation

**Figure 5:**
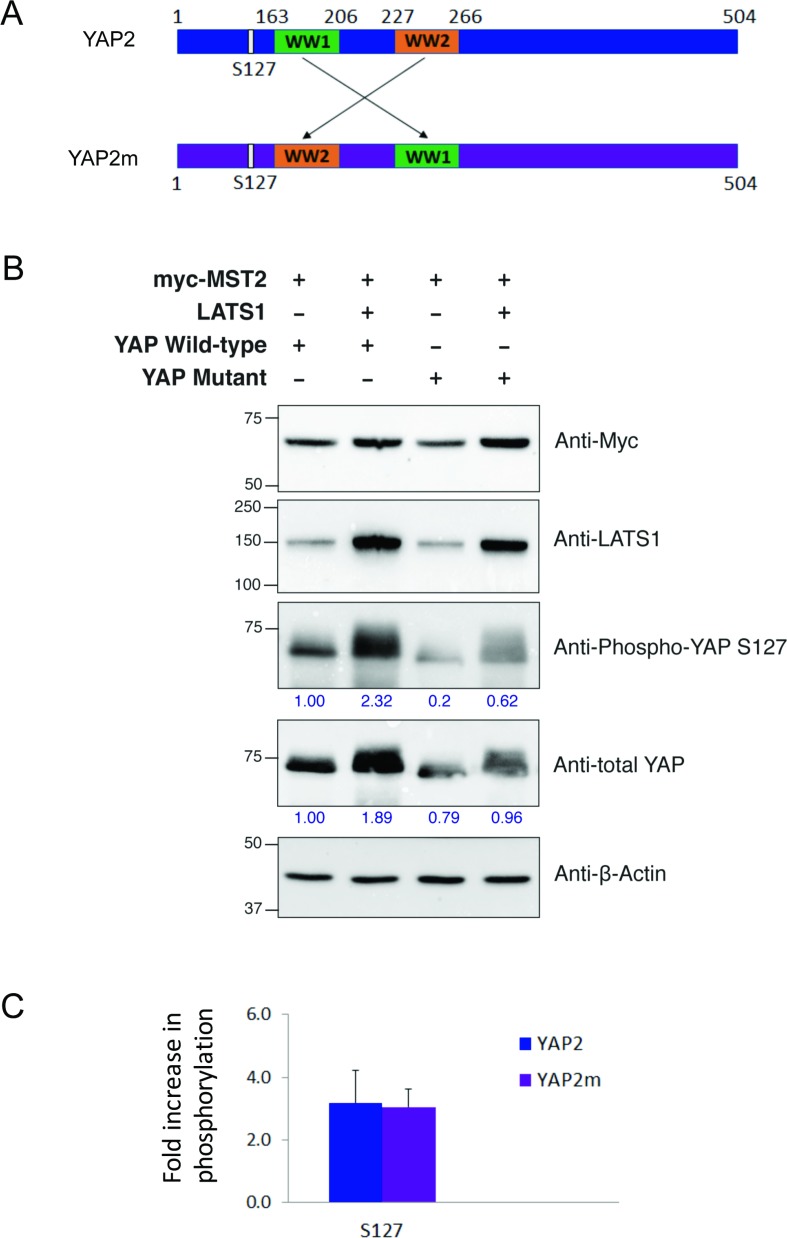
Effect of WW domain swapping in YAP2 on its phosphorylation by LATS1 (**A**) Schematic representations of wildtype YAP2 (blue) and mutant YAP2m (purple) with swapped WW domains. (**B**) HEK293T cells were co-transfected with myc-MST2, LATS1, and either YAP2 wildtype or mutant constructs, as indicated. After 24 h cell lysates were harvested, separated by SDS-PAGE, transferred to membranes, and immunoblotted for myc, LATS1, YAP, phospho-YAP S127, and the loading control β-actin. Size markers are shown in kDa. Band intensities for YAP and phospho-YAP S127 (indicated by numbers in blue) were quantitated using Bio-Rad Image Lab software relative to lane 1. A representative image from one experiment. (**C**) Fold-change increase in phospho-YAP S127 band intensity by the co-transfection of LATS1. Data represents the mean and standard deviation for three independent experiments.

Upon activation by MST kinases, LATS1 phosphorylates YAP2 on several residues, amongst which S127 is highly conserved [27, 28]. Using this site as reference, we tested whether the spatial arrangement of the two WW domains in YAP2 would affect the efficiency of phosphorylation by LATS1. We created a YAP2 mutant, YAP2m in which the two WW domains were swapped (Figure 5A). Upon altering their configuration, the binding of LATS1 and YAP2m might result in the LATS1 kinase domain being differently positioned compared to wildtype YAP2. This would in turn affect the kinase activity of LATS1. We tested this in HEK-293T cells using full length wildtype YAP2 and mutant YAP2m. Interestingly, we observed no significant difference in LATS1 activity, with similar levels of S127 phosphorylation observed for the wildtype and mutant YAP2 (Figure 5B and 5C). From this, we hypothesized that the region between the two PPxY motifs of LATS1 is disordered and may be able to alter its shape depending on its binding partner.

### Region between PPxY motifs in LATS1 is unstructured

**Figure 6:**
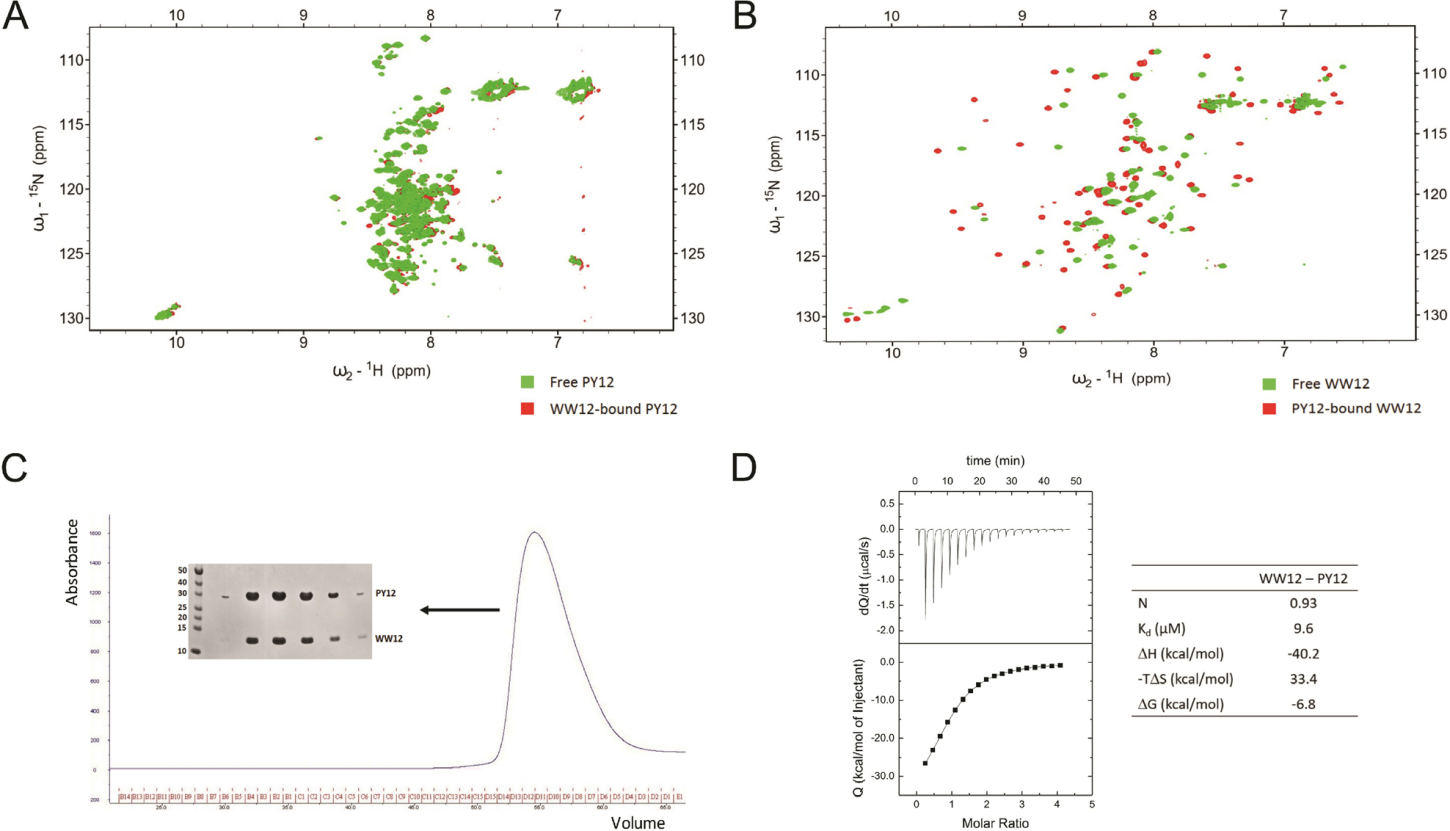
PY12 region from LATS1 is unstructured (**A**) Comparison between HSQC of free PY12 (green) and WW12-bound PY12 (red). (**B**) Comparison between HSQC of free WW12 (green) and PY12-bound WW12 (red) (**C**) Gel filtration elution profile of WW12-PY12 complex, and SDS-PAGE showing WW12 and PY12 at their corresponding molecular weights. Size markers are shown in kDa. (**D**) ITC titration and thermodynamic parameters for the interaction between WW12 and PY12.

We conducted further NMR experiments to examine how the LATS1 binding region changes upon association with its partner. ^15^N-labelled PY12 (containing both PPxY motifs; aa 361–567) was titrated against unlabeled WW12 (containing both WW domains; aa 163–266). The HSQCs of the free and bound PY12 were compared to observe changes in the peaks. The HSQC of free PY12 shows that the protein is unstructured, as most of the peaks are clustered together around the ^1^H chemical shift 8 ppm (Figure 6A green). Upon the addition of the WW12 (final molar ratio of 1:5), there is no significant change in the structure of PY12, with a shift in only a few peaks (Figure 6A red). From our structures, we know that the interacting region between the WW domains and the LATS1 peptides includes only prolines, the tyrosine, and a few residues from the flanking region of the PPxY motif. Prolines are not visible in HSQC and therefore only the remaining few residues appear shifted. This indicates that PY12 interacts with WW12 primarily through the PPxY motif, while the rest of the protein remains unstructured.

Similarly, we repeated the NMR titration with ^15^N-labelled WW12 and unlabelled PY12 to show that the two proteins were interacting with each other. The WW12 HSQC changed significantly upon interaction with PY12 (Figure 6B). This is because several amino acids from both WW1 and WW2 are involved in the interaction with the PPxY motifs, as seen in the structures of the domain-motif complexes.

Finally, when the two constructs (WW12 and PY12) were co-expressed in *E. coli*, they formed a tight complex that could be purified using affinity and gel filtration chromatography (Figure 6C), indicating that the two proteins indeed interacted with high affinity. ITC was performed to determine the thermodynamic parameters of the WW12-PY12 interaction (Figure 6D). The K_d_ was determined to be 9.6 μM, which lies within the range of individual domain-peptide interactions (Figure 1C).

## DISCUSSION

WW domains and their interaction with proteins that contain PPxY motifs has held significant interest in the signaling field for over two decades [29]. WW domains are known to regulate the interactions of numerous proteins and, in some cases, even compete against each other [30]. Studies have shown that tandem WW domains are more selective compared with autonomous WW domains, leading to differential binding properties [31, 32]. Other studies have shown that, in protein complexes with multiple WW domains and PPxY motifs, one of the domain-motif combinations is usually favored, with the other combination(s) acting as additional or supplemental regulators [13, 24]. Indeed, *in vitro* studies show that PPxY peptides bind different WW domains with varying affinities [18, 24, 33].

LATS1 interacts with several WW domain-containing proteins via its PPxY motifs, including the Hippo pathway effector, YAP. Our ITC data showed that WW1 of YAP2 preferentially binds PPxY2 from LATS1 and vice-versa, suggesting that WW1-PPxY2 binding forms the basis of the interaction, with additional regulation from WW2-PPxY1 binding. Once the first attachment is established between WW1 and PPxY2, the WW2-PPxY1 interaction would be further favoured and its binding affinity would increase, since the roto-translational entropic penalty would be lower. Also, the WW1 domain is conserved among the other isoforms of YAP that contain only a single domain, indicating that WW1 is functionally more important for YAP interactions, making it a potential target for drug design.

Mutation of key residues from YAP2 WW1 revealed that leucine (L190) and histidine (H192) are important for binding. Sequence alignment of WW domains from other proteins [23] shows that L190 is usually replaced by either isoleucine or valine while H192 is highly conserved in other WW domains. We can therefore extrapolate that these conserved residues play a key role in the interaction of WW domains with PPxY peptides. Mutation of residues flanking the PPxY motif did not affect their binding affinity, suggesting that other residues from the motif may be involved. Studies on alanine-scanning mutagenesis of PPxY-containing peptides from ErbB4 also indicated that flanking residues do not affect its binding with WW domains dramatically [34]. The authors [34] proposed that the non-consensus residues may not be required for driving their interaction with WW domains but may play a role in stabilizing the conformation of PPxY peptides.

Recently, structures of WW domains in complex with PPSY and PPCY motifs were reported [24, 35]. We compared these structures with our WW1-PPPY2 structure to examine the differences due to the presence of a different amino acid in PPxY. In both cases, the binding affinity between the domains and peptides was 3 to 4 μM. Interestingly, the conformation of the peptides changed from PPII to 3_10_ helical conformation, following the S/C residues (Supplementary Figure 6). The structural alignment shows that the side chains of these residues are present in a very similar conformation (Supplementary Figure 6). In case of S, there is a hydrogen bond with i+3 residue that stabilizes the 3_10_ helix. This further suggests that in addition to recognition, these residues play a role in stabilizing the peptide fold.

**Figure 7:**
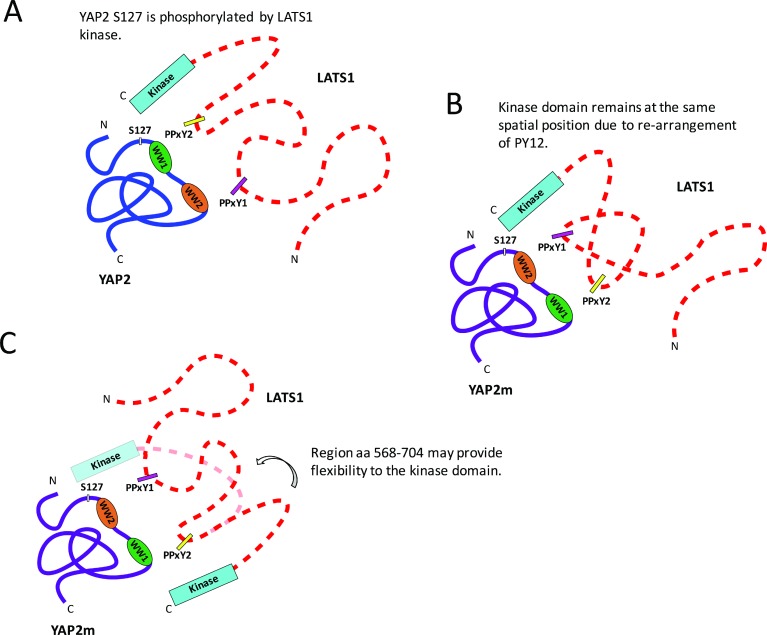
Schematic representation to show the proposed models for the interaction between YAP2 and LATS1 YAP2: blue; YAP2m: purple; LATS1: dotted red; WW1: green; WW2: orange; PPxY1: magenta; PPxY2: yellow. (**A**) LATS1 interacting with wildtype YAP2 via PPxY motif-WW domain interactions, resulting in phosphorylation at S127. (**B**) LATS1 interacting with YAP2m, containing swapped WW domains. The region between the PPxY motifs in PY12 is unstructured and might re-arrange depending on the orientation of WW domains, thereby maintaining the same spatial configuration of LATS1 kinase domain as wildtype YAP2. (**C**) Alternatively, the spatial position of LATS1 kinase domain may change upon interaction with YAP2m. However, the LATS1 region between PY12 and kinase domain (aa 568-704) may be flexible, allowing the kinase domain to phosphorylate different regions of YAP2.

Although the role of YAP as a substrate of LATS1 is well documented [16, 17], the molecular basis for their interaction is not fully understood. Secondary structure predictions from the protein sequence indicate that LATS1 is largely disordered except for its C-terminal kinase domain. Our NMR experiments also confirm that the region between the PPxY motifs (PY12) is unstructured, even upon binding to its partner. Furthermore, we found that LATS1-mediated S127 phosphorylation is not affected by swapping the two WW domains in YAP2. It is therefore possible that there is no directionality between the domains and motifs of YAP2 and LATS1, and that a specific orientation of the WW domains is not required for LATS1 binding and phosphorylation at S127. However, this possibility is less likely because of the differential binding affinities of the WW domains-PPxY peptides. Although the YAP2 mutant should be tested for other phosphorylation sites in YAP2, based on our observations, we make two speculations as to the binding. First, because the region between the PPxY motifs (PY12) of LATS1 is unstructured, the motifs may rearrange themselves as per the orientation of the WW domains, thereby maintaining the correct spatial configuration of the LATS1 kinase domain and not interfering with its phosphorylation activity (Figure 7B). Second, the LATS1 region between PY12 and the kinase domain (aa 568-704) may be flexible, allowing the kinase domain to access different regions of its binding partner (Figure 7C).

In summary, our studies reveal that YAP2 WW domains prefer different LATS1 PPxY motifs *in vitro*. Besides the conserved tryptophan, leucine (L190) and histidine (H192) are conserved residues in WW domains and play critical roles in their interaction with PPxY motifs. There is a putative functional conservation of S127 phosphorylation of YAP2 by LATS1 kinase that is independent of the configuration of the WW domains in the YAP2 and LATS1 complex. LATS1 is mainly unstructured, and this property may regulate its interactions with different binding partners.

## MATERIALS AND METHODS

### Cloning, expression and purification of proteins

The YAP2 sequences WW1 (aa 163-206), WW2 (aa 227-266), WW12 (containing both WW domains; aa 163-266), WW1-4gly-PPxY2, WW2-4gly-PPxY1 and all WW1 mutants were cloned into pGEX-6P-1. LATS1 region PY12 (containing both PPxY motifs; aa 361-567) was cloned into pACYC. Full-length LATS1, YAP2, and YAP2m, with swapped WW domains (residues mentioned above) were cloned into pcDNA3.1(-).

Constructs were transformed into *E. coli* BL21 (DE3) cells and grown in LB media at 37°C to OD_600_ 0.6 to 0.8. Protein expression was induced by the addition of 0.2 mM IPTG and the cultures were grown at 20°C overnight. For ^15^N- and ^13^C-labelled samples, cultures were grown in M9 media containing ^15^NH_4_Cl and ^13^C-glucose.

Cells were harvested by spinning at 4,000 *g* for 20 min and lysed by sonication in buffer containing 50 mM Tris-HCl pH 7.0, 200 mM NaCl, 5% glycerol, 2 mM DTT, 0.5% Triton X-100 and 1mM PMSF. The lysates were centrifuged at 30,000 *g* for 30 min at 4°C and the supernatant was subjected to GST affinity chromatography. The GST tag was cleaved overnight using PreScission Protease. In the case of WW12-PY12 complex, an additional Ni-NTA chromatography step was performed, as PY12 contained a His-tag. The eluted proteins were further purified using FPLC Superdex 75 gel filtration in buffer containing 20 mM Tris-HCl pH 7.0, 100 mM NaCl, 5% glycerol and 2 mM DTT. The purity and quality of the purified proteins were assessed using SDS polyacrylamide gel electrophoresis (SDS-PAGE) and dynamic light scattering (DLS).

### Isothermal titration calorimetry (ITC)

ITC experiments were performed using the MicroCal VP-ITC titration calorimeter (MicroCal-Malvern). The LATS1 peptides containing PPxY motifs, NRQPPPPYPLTA (PPxY1; aa 369-380) and NYQGPPPPYPKH (PPxY2; aa 551-562) and their mutants, NAQPPPPYPLTA (PPxY1m) and NYAGPPPPYPKH (PPxY2m), were obtained from GL BioChem. The proteins and peptides used were prepared in the same buffer containing 20 mM Tris-HCl pH 7.0 and 100 mM NaCl. Protein concentrations varying from 20 to 70 μM were titrated against peptide concentrations ranging from 0.5 to 1.8 mM at 25°C. The peptide samples were also injected into buffer as a reference to estimate the heat of dilution. In the case of WW12 vs PY12, the buffer used was 20 mM Tris-HCl pH 7.0, 100 mM NaCl, 3% glycerol, 2 mM DTT and 2 mM EDTA. All samples were degassed and spun before the experiment. The binding isotherms were analyzed employing a model that considered a single ligand binding site implemented in Origin 7 (OriginLab).

### Linking WW domains with PPxY peptides

The linked complexes of WW1-PPxY2 and WW2-PPxY1 were prepared using a poly-glycine linker. The glycines were inserted using fusion PCR by designing primers containing glycine sequence overhangs. Several lengths were tested, varying from 4 to 8 glycines. The optimum length was determined by comparing the elution profiles from analytical Superdex 75 gel filtration column.

### NMR structure determination

The domain-peptide linked constructs labelled with ^15^N and ^13^C were exchanged into buffer containing 20 mM sodium phosphate pH 6.0 and 100 mM NaCl. The samples were concentrated to 1 mM and D_2_O was added to a final concentration of 5%. Data collection was performed at 25°C using a Bruker Avance 800 MHz spectrometer equipped with a TXI cryogenic probe. ^1^H, ^15^N, and ^13^C resonance assignments were obtained from 3D HNCACB, 3D CBCA(CO)NH [36], and 3D HCCH-TOCSY spectra [37]. Inter-proton distance restraints for structure calculation were assigned from 3D ^15^N-edited NOESY-HSQC, 3D ^13^C NOESY-HSQC, and 2D ^1^H-^1^H NOESY spectra using a 100 ms mixing time. All spectra were processed by NMRPipe/NMRDraw [38] and data analysis was done using Sparky [39]. Dihedral angle restraints were predicted by the TALOS program [40]. The solution structures (100) were calculated using the Xplor-NIH 2.24 software package [41], using 10,000 steps of simulated annealing and ensembles of the 20 lowest energy structures were selected.

### NMR titration

WW12 and PY12 were individually labelled with ^15^N and purified in buffer containing 20 mM Tris-HCl pH 7.0, 50 mM NaCl, 3% glycerol, 5 mM DTT and 2 mM EDTA. The samples were concentrated ranging from 400 to 500 μM, and D_2_O was added to a final concentration of 5%. The labelled samples were then titrated against their unlabelled partners to a final molar ratio of 1:5. The HSQC spectra were measured as mentioned previously.

### Cell culture and transfection

HEK-293T cells (purchased from ATCC) were grown in DMEM medium (Nacalai Tesque), supplemented with 10% fetal bovine serum (FBS, HyClone) and 100U/mL penicillin/streptomycin (Nacalai Tesque).

Sub-confluent 60 mm dishes of HEK-293T cells were co-transfected with 1.3 μg of pCI-neo-myc-hMST2 (a gift from Wanjin Hong and Lim Yoon Pin), 1.3 μg of pcDNA3.1(-)-YAP2 wildtype or mutant, 1.3 μg of pcDNA3.1(-)-LATS1, and 1.3 μg of pcDNA3.1(-)-empty vector as required to make the total DNA to 3.94 μg, using TransIT-293 Transfection Reagent (Mirus) according to the manufacturer's specifications. After 24 h, cells were lysed in 400 μL of immunoprecipitation buffer (50 mM Tris pH 8.0, 150 mM NaCl, 1% (v/v) NP-40 containing 1:100 protease inhibitor cocktail (Sigma-Aldrich) and 1:100 phosphatase inhibitor cocktail (Sigma-Aldrich). Samples were rotated at 4°C for 1 h before centrifugation at 16,000 g for 10 min. Lysates were quantitated using Bio-Rad Protein Assay Reagent.

### Western blotting

Protein lysates (30 μg of total protein) were boiled in sample buffer (2% (w/v) SDS, 10% (v/v) glycerol, 62.5 mM Tris pH 6.8, 0.02% (w/v) bromophenol blue, with 1% (v/v) 2-mercaptoethanol) and separated by SDS-PAGE using linear Tris-glycine gels in running buffer (25 mM Tris pH 8.3, 192 mM glycine, 0.1% SDS (w/v)) using the Mini-PROTEAN Tetra Cell system (Bio-Rad). The Bio-Rad Precision Plus Protein All Blue Pre-Stained Standards (Cat. 161-0373) was used as the molecular weight reference.

Separated proteins were transferred to Immun-Blot PVDF membranes (Bio-Rad Cat. 162-0177) using the Mini Trans-Blot Cell system (Bio-Rad) at 90 V for 90 min at 4°C in transfer buffer (25 mM Tris-base, 192 mM glycine, 20% (v/v) methanol). Membranes were then washed with Tris- buffered saline containing Tween-20 (TBST; 25 mM Tris pH 7.5, 150 mM NaCl, 0.1% (v/v) Tween-20) and then blocked with 5% (w/v) skim milk powder in TBST for 30 min at room temperature. Blotting was performed with primary antibodies overnight at 4°C. Membranes were washed four times for 5 min each with TBST and then incubated with secondary antibody for 1 h at room temperature, before washing again with TBST. Signals were detected using the Bio-Rad ChemiDoc Touch Imaging System with WesternBright Quantum Western Blotting HRP Substrate (Cat. K-12042-D10). Band intensity was quantitated using Bio-Rad Image lab software.

### Antibodies

The following antibodies were used: anti-myc (Santa Cruz Biotechnology, Cat. sc789), anti-LATS1 (Abcam, Cat. ab70561), anti-phospho-YAP S127 (Abcam, Cat. ab76252), anti-YAP (made in house as described in [17]), and Anti-β-Actin (Cell Signaling Technology, Cat. 3700).

## SUPPLEMENTARY MATERIALS FIGURES


