# Supplementary material for: Biophysical studies and NMR structure of YAP2 WW domain - LATS1 PPxY motif complexes reveal the basis of their interaction
Source: Oncotarget. 2018 Jan 3;9(8):8068–80. doi: 10.18632/oncotarget.23909 (PMC5814282; doi:10.18632/oncotarget.23909)
Supplement: Supplementary file 1 [file oncotarget-09-8068-s001.pdf]

Biophysical studies and NMR structure of YAP2 WW domain-LATS1 PPxY motif complexes reveal the basis of their interaction

SUPPLEMENTARY MATERIALS

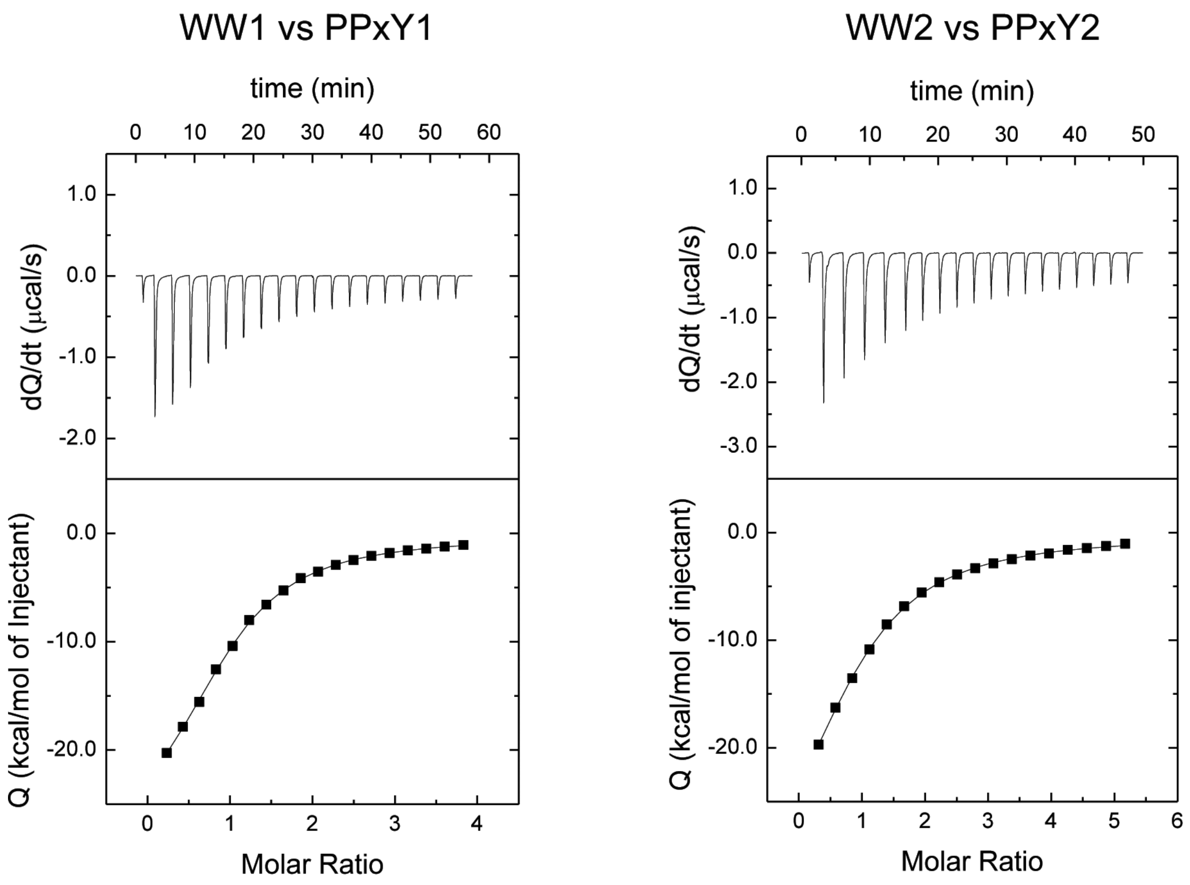

Supplementary Figure 1: ITC isotherms for WW1-PPxY1 and WW2-PPxY2. These domain-motif combinations exhibited the lowest binding affinities.

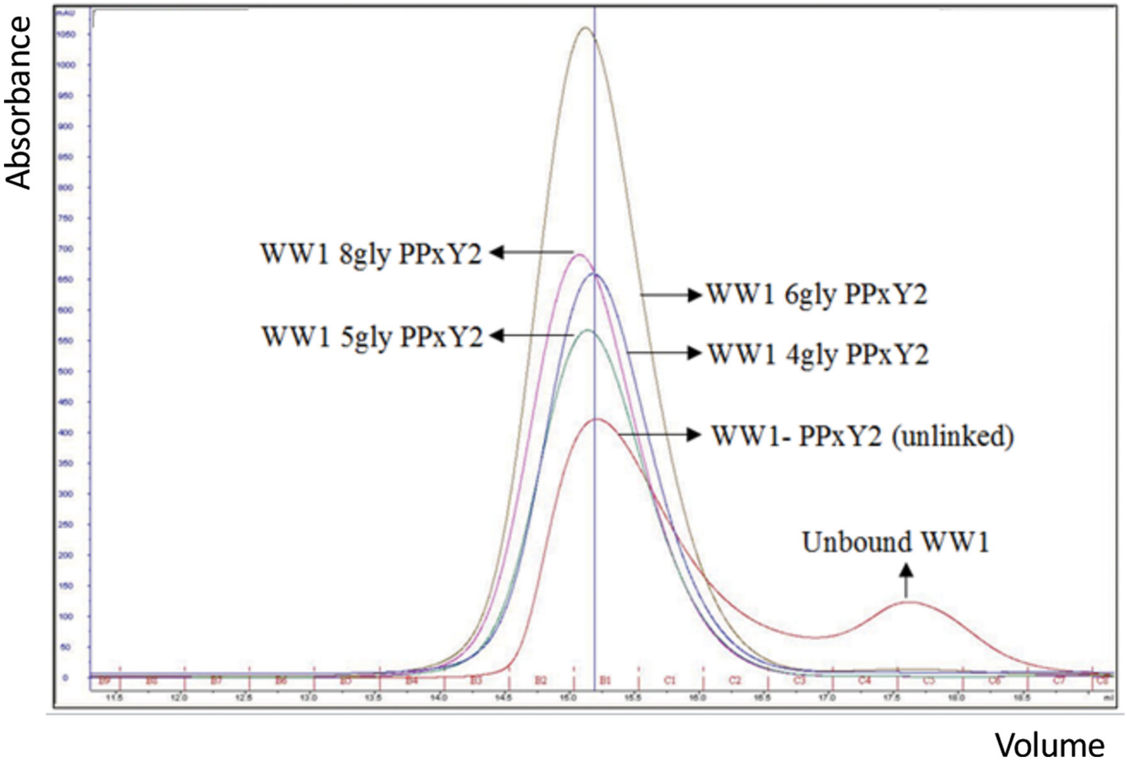

Supplementary Figure 2: Linking of WW domain to PPxY peptide. Comparison of gel filtration elution profiles of different lengths of glycine linkers (4-8 glycine residues) using analytical Superdex 75 column. Unlinked WW1-PPxY2 and WW1-4gly-PPxY2 elute at the same volume (15.2 ml), whereas longer linkers elute at slightly larger volumes. A fraction of the unlinked complex dissociated during gel filtration giving a separate WW1 peak (at 17.5 ml).

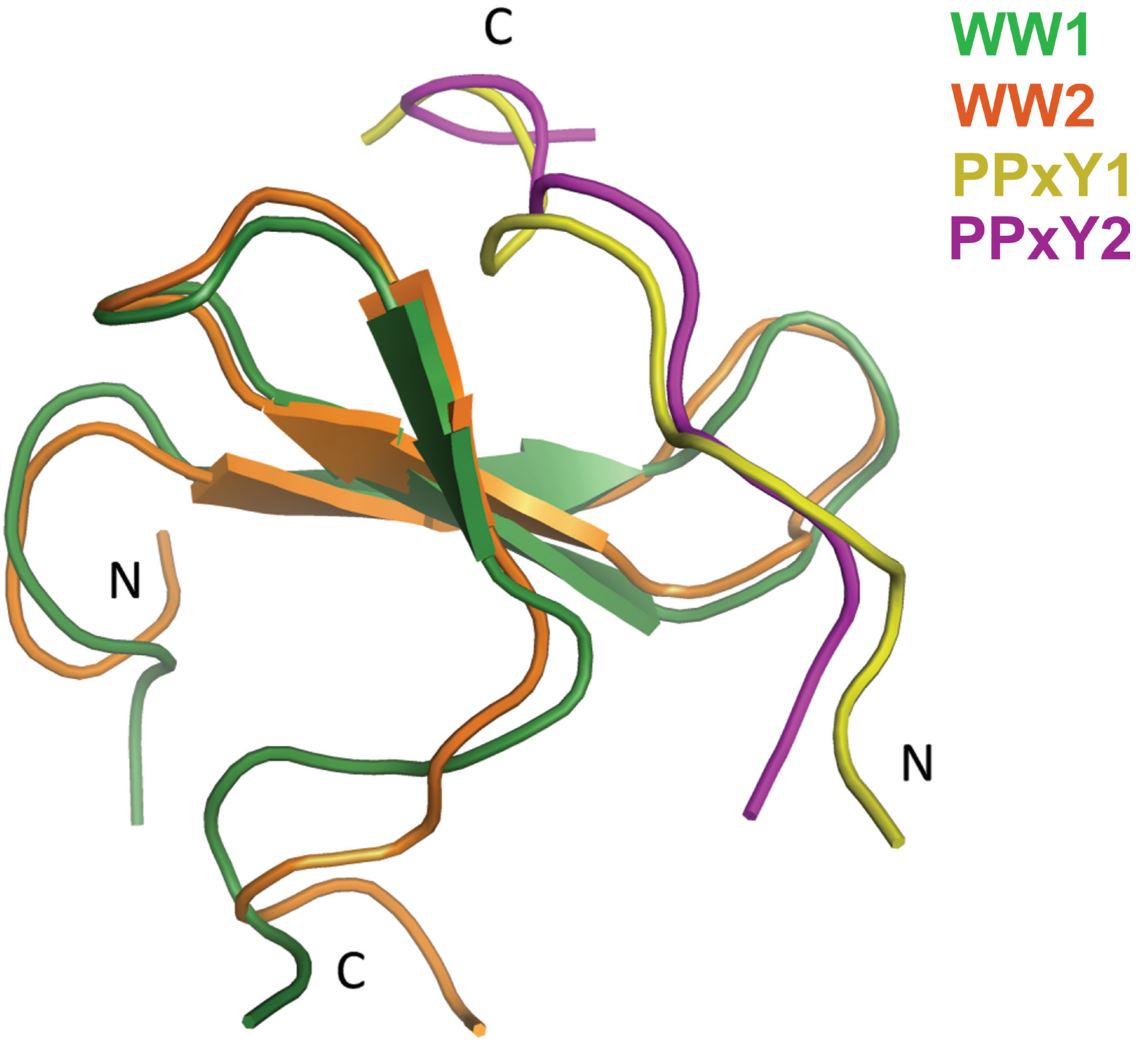

Supplementary Figure 3: Superimposition of WW1-PPxY2 and WW2-PPxY1 structures. Structural alignment of the two WW domains of YAP2 in complex with LATS1 PPxY peptides (WW1: green, PPxY2: yellow, WW2: orange, PPxY1: magenta), showing the similarity between them. The RMSD between structures is 1.2Å.

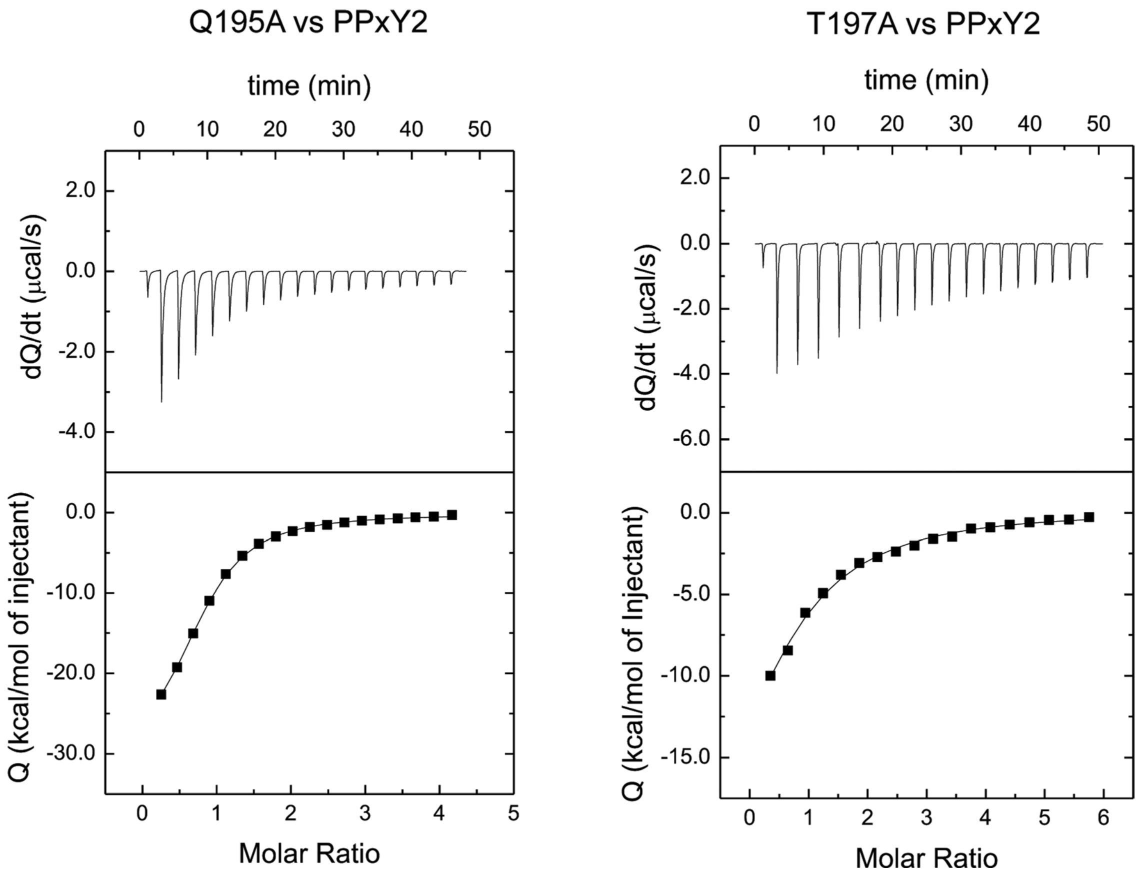

Supplementary Figure 4: ITC isotherms for WW1 mutants, Q195A and T197, with PPxY2. These WW1 mutants reduced the affinity by a smaller fraction compared to L190 and H192 (Figure 4A and 4B).

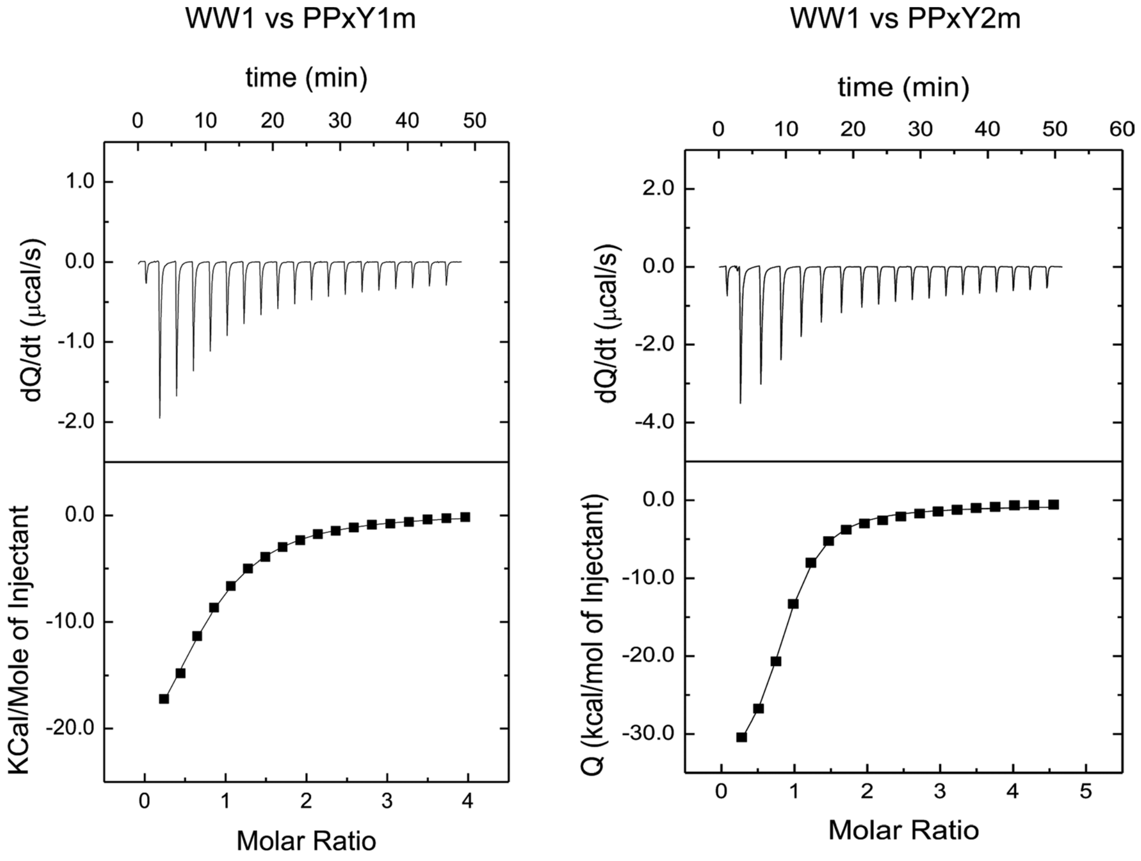

Supplementary Figure 5: ITC isotherms for PPxY2 mutants with WW1 domain. These mutants from flanking residues of the PPxY motif did not affect the binding affinity (Figure 4C) with WW1.

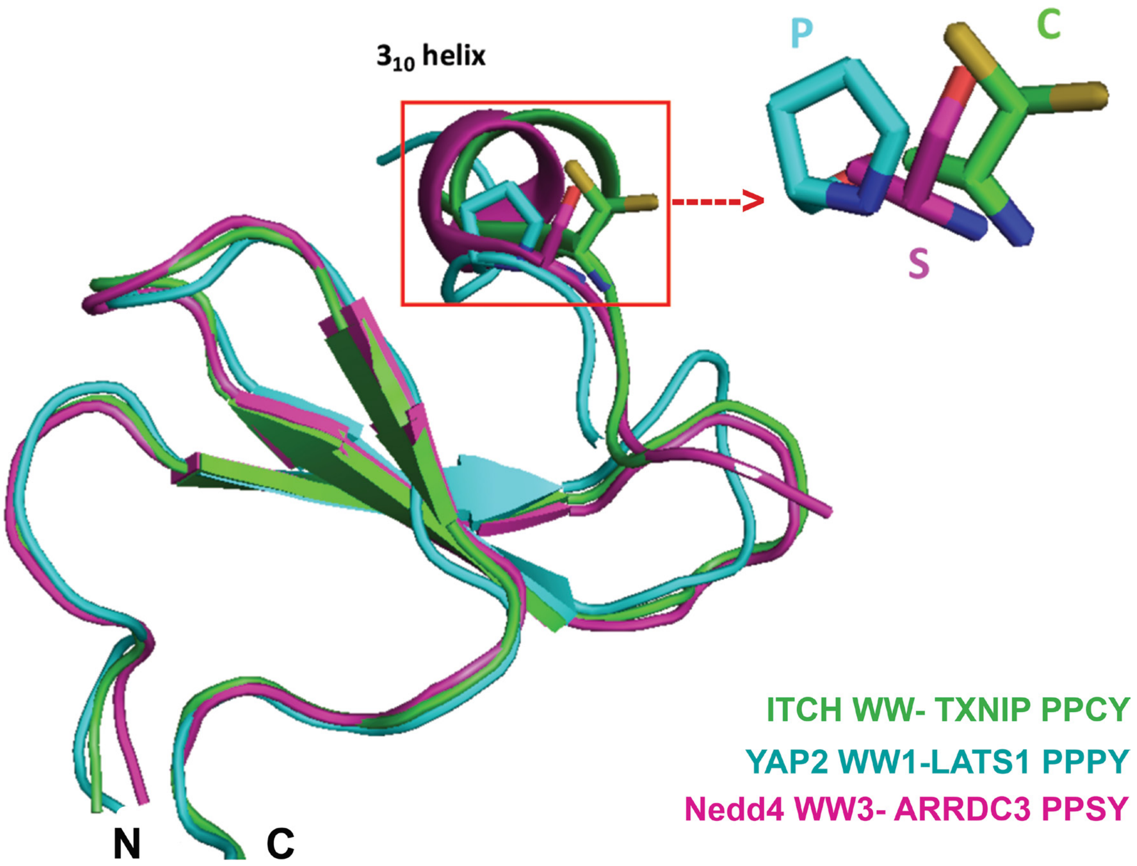

Supplementary Figure 6: Comparison of WW domain structures with other PPxY motifs. Structural alignment of YAP2 WW1-LATS PPPY (cyan) with Nedd4 WW3-ARRDC3 PPSY (magenta) (PDB id 4N7H) and ITCH WW-TXNIP PPCY (green) (PDB id 4ROH). The side chains of P, S, and C (red box) from the PPxY motifs are shown. The side chains are present in similar configurations, with S (magenta) and C (green) involved in 3_{10} helix formation.
